# Biomarkers of cellular senescence and major health outcomes in older adults

**DOI:** 10.1007/s11357-024-01474-9

**Published:** 2024-12-18

**Authors:** Steven R. Cummings, Li-Yung Lui, Aversa Zaira, Theresa Mau, Roger A. Fielding, Elizabeth J. Atkinson, Sheena Patel, Nathan LeBrasseur

**Affiliations:** 1 San Francisco Coordinating Center, California Pacific Medical Center Research Institute, San Francisco, CA USA; 2 Department of Epidemiology and Biostatistics, University of California, San Francisco, CA USA; 3 Robert and Arlene Kogod Center on Aging, Paul F. Glenn Center for the Biology of Aging Research, and Department of Physical Medicine and Rehabilitation, Mayo Clinic, Rochester, MN USA; 4 Metabolism and Basic Biology of Aging Directive, Jean Mayer USDA Human Nutrition Research Center on Aging, Tufts University, Medford, MA USA; 5 Department of Quantitative Health Sciences, Mayo Clinic, Rochester, MN USA

**Keywords:** Geroscience, Cell senescence, Senescence associated secretory phenotype, Biomarkers

## Abstract

The geroscience hypothesis proposes that underlying biological processes, such as the accumulation of senescent cells, have deleterious effects on multiple tissues and increase the risk of many chronic conditions with aging. Senescent cells produce heterogenous biomarkers, also called senescence-associated secretory phenotype (SASP). Circulating concentrations of senescence biomarkers may reflect an underlying burden of senescent cells in various tissues. Plasma levels of these proteins have been associated with increased mortality and poorer physical function. The associations of them with the incidence of major age-related conditions including heart failure, cardiovascular disease, stroke, and dementia, have not been studied. We measured 35 senescence biomarkers in baseline plasma samples from 1678 participants aged 70–79 years old in the longitudinal Health ABC cohort study. Clinical outcomes were ascertained and validated over an average 11.5 year follow-up. In models adjusted for age, sex, and race, higher levels of most of senescence biomarkers were associated with increased risk of all-cause mortality, mobility limitation, and heart failure. Several were also associated with an increased risk of coronary heart disease, stroke, and dementia. Very few were associated with the risk of cancer. Proteins that were selected by Lasso regression for each outcome that commonly included GDF15 and IL6, significantly improved the prediction of mortality, mobility limitation, and heart failure compared with age, sex, and race alone. These results indicate that levels of senescence biomarkers predict an increased risk of several age-related clinical outcomes and may identify individuals most likely to benefit from senotherapeutics.

**Supplementary Information:**

The online version contains supplementary material available at 10.1007/s11357-024-01474-9.

## Introduction

Clinical manifestations of aging include functional deficits, chronic diseases, and geriatric syndromes. The emergence and co-occurrence of such conditions in later life suggests that aging itself is a root cause of their pathogenesis. This premise has led to the geroscience hypothesis that interventions that target biological mechanisms of aging may delay if not prevent prevalent age-associated diseases and disabilities as a group [1].

In support of the geroscience hypothesis, studies in model organisms have demonstrated the therapeutic benefits of interventions targeting mechanisms of aging. One example is cellular senescence, a cell fate in response to diverse forms of stress and damage that is often characterized by growth arrest, activation of pro-survival pathways, and secretion of robust and heterogenous biomarkers of cell senescence also known as senescence-associated secretory phenotype (SASP) [2]. Senescent cells accumulate with advancing age and are enriched in tissues that are susceptible to age-associated diseases [3]. In mouse models of aging, genetic and pharmacologic approaches (senotherapeutics) to selectively eliminate senescent cells (senolytics) or suppress their SASP (senomorphics) have been shown to counter deleterious changes in the function of the heart, lungs, brain, kidney, skeletal muscle, and other organs [4–10]. These studies have generated enthusiasm for the potential of senescent cell-targeting interventions to extend healthy life years and compress morbidity in humans.

In addition to mediating the biological effects of senescent cells locally, components of the SASP can enter the circulation and act distally [11–14]. Correspondingly, the concentration of these factors in the blood may reflect senescent cell burden in various tissues. Our group and others have studied the associations between candidate senescence biomarkers—particularly circulating concentrations of cytokines, chemokines, matrix remodelers, growth factors, and other protein components the biomarkers of senescence—and the incidence of health outcomes including tests of physical performance, frailty, and all-cause mortality in separate human cohorts [15–17]. Potential association between higher level senescence biomarkers and the risk of diseases whose incidence increases with aging, such as cardiovascular disease, stroke, dementia, and cancer, have not been examined. We used archived serum and data from the prospective Health ABC cohort study to assess the association of these senescence biomarkers with major health outcomes in older adults.

## Methods

Health ABC is a prospective cohort study of 3075 community dwelling older adults aged 70 to 79 years who were recruited and examined in 1997 and 1998 from a list of Medicare beneficiaries residing near Pittsburgh, PA, and Memphis, TN. The goal was to enroll similar numbers of black and whites and females and males. These participants were contacted by telephone every 6 months and attended clinical visits annually or biannually until 2015. During these follow-ups, health status was assessed, occurrence of major diseases such as coronary heart disease (CHD), heart failure (HF), and stroke, and data about interim hospitalizations or major outpatient procedures were collected and centrally adjudicated. All participants provided written informed consent and IRB approval was obtained for each clinical site and coordinating center. This present study includes 1678 participants randomly selected from the baseline examination.

### Outcomes

Outcomes were collected during follow-up for a mean of 11.5 years and up to 16.6 years. Deaths were identified by an adjudication committee reviewing information from scheduled follow-up contacts, medical records, obituaries, death certificates, and files from the Centers for Medicare and Medicaid Services. Participants were asked every 6 months about ability and difficulty in walking ¼ mile or climbing 10 steps without resting and mobility limitation was defined as severe mobility limitation: a lot of difficulty or inability to walk one-quarter of a mile or climb 10 steps at two consecutive assessments [18]. All potential cases of HF were reviewed and adjudicated by study investigators. Ascertainment of HF rested on diagnosis by a physician, presence of supportive symptoms and signs, pharmacotherapy involving a diuretic and digitalis or a vasodilator, or documented evidence from diagnostic imaging such as cardiomegaly and pulmonary edema on chest X-ray, or cardiac dilatation or dysfunction on echocardiography or ventriculography. CHD was defined as myocardial infarction, angina, coronary artery angioplasty, coronary bypass surgery, CHD death, or revascularization by a panel of physicians as previously described. Potential cases of stroke were also reviewed and adjudicated by study investigators; we did not differentiate ischemic and hemorrhagic strokes for this analysis. Dementia was determined using an algorithm that considered if participants were prescribed dementia medications, had hospital records with dementia as a discharge diagnosis, or had a decline in the 3MSE score of more than 1.5 SD from baseline [19]. Incident cancer cases were identified by self-report and review of outpatient and hospitalizations and confirmed by pathology reports.

### Biomarkers of cellular senescence

We initially identified candidate senescence biomarkers based on published reports of SASP components that are expressed and/or change in abundance in parallel with other markers of senescence in tissues. The list of candidates was refined through the analysis of the secretome of several human cell types driven to senescence in vitro [17], their inclusion in senescence-focused gene sets [20, 21], and evidence that their circulating concentrations were affected by the induction or clearance of senescence cells in mice [22–24].

In the present study, 35 senescence-related proteins were measured in serum samples that had been obtained at the baseline Health ABC examination and archived at − 70° (full and alternative names listed in Supplemental Table 1). The concentrations of ADAMTS13, Eotaxin, Fas, GDF15, GRO$\alpha$, ICAM1, IL6, IL7, IL8, IL10, IL15, IFN$\alpha$, MCP1, MDC, MMP1, MMP7, MMP9, MPO, OPN, PAI1, PARC, PDGF-AA, PDGF-AB, RAGE, RANTES, SOST, STC1, TARC, TNFR1, TNFR2, TNFα, TRAIL, uPAR, and VEGFA were determined with commercially available multiplex magnetic bead-based immunoassays (R&D Systems) on the Luminex xMAP multianalyte profiling platform and analyzed on MAGPIX System (Merck Millipore) according to the standard manufacturer’s protocols. Activin A concentration was measured using a Quantikine ELISA Kit (R&D Systems) according to the manufacturer’s specifications. Assay performance parameters have been reported previously [25].

**Table 1:** Characteristics of participants

Number	1678
Age, years, mean + / − SD	73.6 + / − 2.9
Gender	
Male, *n* (%)	803 (47.9)
Female, *n* (%)	875 (52.1)
Race	
White, *n* (%)	968 (57.7)
Black, *n* (%)	710 (42.3)
BMI (kg/m^2^, mean + / − SD)	27.4 ± 4.8
History of coronary heart disease, *n* (%)	353 (21.0)
History of heart failure, *n* (%)	53 (3.2)
History of stroke, *n* (%)	158 (9.4)
History of cancer, *n* (%)	279 (16.6)
History of dementia, *n* (%)	0 (0)
History of mobility limitation, *n* (%)	0 (0)

### Statistical methods

Descriptive statistics of demographics and prevalent cases were summarized as mean (SD) or number (%). Spearman correlations between the serum concentrations of all proteins and age are presented in a heat map. We used multivariable Cox proportional hazard models to examine the relationship between the senescence biomarkers and time-to-event outcomes using proteins quartiles with the lowest quartile as the reference group, adjusted for age, sex, and race. To provide a visual summary of the associations of numerous biomarkers and clinical outcomes, we display statistically significant associations between the clinical outcomes and all biomarkers that had at least one significant association with one of the outcomes.

We then applied least absolute shrinkage and selection operator (Lasso) regression to the biomarkers for each outcome and then included those factors with significant coefficients in multivariable Cox proportional hazard models. When applying Lasso regression, any biomarker that was skewed distributed was log transformed, if appropriate, winsorized, and then standardized by subtracting the mean and dividing by the standard deviation. Cross-validation with Harell *C* index was used to select optimal tuning parameter (minimum lambda).

*C*-statistics were calculated to assess model performances using 1) age, sex, and race; 2) all biomarkers with significant coefficients from Lasso regression only; and 3) age, sex, and race, along with the markers. We used bootstrapping to calculate the 95% confidence interval for each *C*-statistics and compared *C*-statistics between 3 models. Analyses were conducted in SAS version 9.4 and R studio version 4.05.

## Results

The 1678 Health ABC participants included in this study had an average age of 73.6 years, with 52.1% being female and 42.3% self-reporting as black (Table 1). Over the course of an average follow-up period of 11.5 years, there were 371 incident cases of coronary heart disease, 360 incident cases of heart failure, 154 incident cases of stroke, 331 cases of dementia, 356 incident cases of non-skin cancer, 1314 cases of mobility limitation, and 1030 deaths.

Supplemental Tables 2–8 list the senescence biomarkers that were tested for associations with aging-related conditions. In a heat map of correlations between the senescence biomarkers, they were, in general, weakly, or not correlated with each other (Supplemental Figure S1). Correlations with age were weak; however, the baseline age range for Health ABC participants (70–79 years old) was narrow.

**Table 2:** Summary of the *C*-statistics (95% CI) for outcomes with models adjusted for clinical covariates and clinical covariates with serum concentrations of senescence biomarkers

Outcome	Model 1 (age, sex, race adjusted)	Significant senescence biomarkers included	Model 2 (model 1 + senescence biomarkers)
Mortality	0.61 (0.59, 0.62)	GDF15, IL6, Eotaxin, MMP1, MMP7, Activin A, TRAIL	0.68 (0.66, 0.69)
Mobility limitation	0.58 (0.57, 0.60)	TNFR1**,** GDF15, IL6, MPO, Eotaxin	0.66 (0.64, 0.67)
Heart failure	0.59 (0.56, 0.62)	GDF15, PARC, MMP7	0.70 (0.68, 0.73)
Coronary heart disease	0.59 (0.56, 0.62)	GDF15, IL6	0.65 (0.63, 0.68)
Stroke	0.59 (0.54, 0.63)	Eotaxin, IL6	0.67 (0.63, 0.72)
Dementia	0.63 (0.60, 0.66)	GDF15, MMP1	0.68 (0.65, 0.71)
Cancer	0.61 (0.58, 0.64)	MMP1, Activin A, TRAIL, uPAR	0.64 (0.61, 0.67)

### Patterns of association between senescence biomarkers and disease risk

For almost all associations, higher serum concentrations of senescence biomarkers were associated with an increased risk of the aging outcome. Five biomarkers, GDF15, IL6, MMP1, MMP7, and TNFR2, were significantly associated with all 6 non-cancer aging-related conditions, mortality, mobility limitation, heart failure, coronary heart disease, stroke, and dementia. All of the aging-related conditions were associated with at least several of the biomarkers except cancer which was associated with very few senescence biomarkers. Quantitative details of the associations between all senescence biomarkers and the outcomes are in Supplemental Tables 2–8.

### The association between senescence biomarkers and mortality and mobility limitations

Twenty-six of the 35 senescence biomarkers were significantly associated with an increased all-cause mortality. These included GDF15, VEGFA, PARC, MMP2, and TNFR1. Consistent with previous studies that used objective measures of physical function [15], we observed significant associations between 26 biomarkers of cellular senescence and self-reported mobility limitation (Supplemental Table S3).

### The association between senescence biomarkers and major age-related diseases

For cardiovascular diseases, we observed significantly increased risk of incident heart failure (HF) using 20 senescence biomarkers when comparing participants in Q4 to Q1 (Supplemental Table S4). GDF15, IL6, TNFR1, MMP7, and PARC demonstrated the highest HRs for HF. Participants in Q4 compared to Q1 of 14 senescence biomarkers demonstrated significantly increased risk for incident coronary heart disease (CHD) (Supplemental Table S5). Participants in Q4 of GDF15, IL6, Activin A, MCP1 exhibited the highest HRs (all ≥ 1.6) for CHD relative to Q1 participants.

We observed that participants in Q4 of nine senescence biomarkers had increased risk of stroke in the follow-up period compared to participants in Q1 (Supplemental Table S6). Similar to CHD and HF, higher concentrations of GDF15 and IL6, along with TNFR1, Eotaxin, MMP1, and MMP2, conferred the highest HRs for incident stroke.

For dementia, 12 individual senescence biomarkers had significantly greater HRs comparing participants in Q4 with participants in Q1 (Supplemental Table S7). The greatest risk was conferred by the highest quartiles of GDF15, TNFR1, MMP1, MMP7, and µPAR, with participants in Q4 for each biomarker having a HR ≥ 1.6 for incident dementia relative to those in Q1.

Relative to CHD, HF, stroke, and dementia, few senescence biomarkers were associated with incident cancer (Supplemental Table S8). Only Health ABC participants in the highest compared to the lowest quartiles of MMP1, Activin A, and OPN at baseline demonstrated significantly increased risk for cancer during follow-up with HR ≥ 1.4 for MMP1 and OPN.

**Fig. 1:**
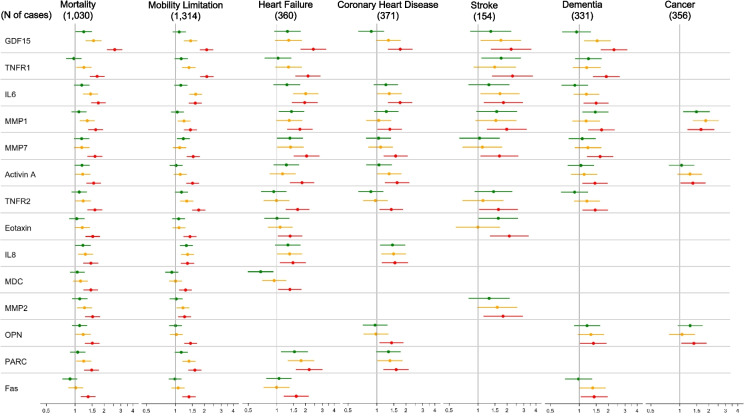

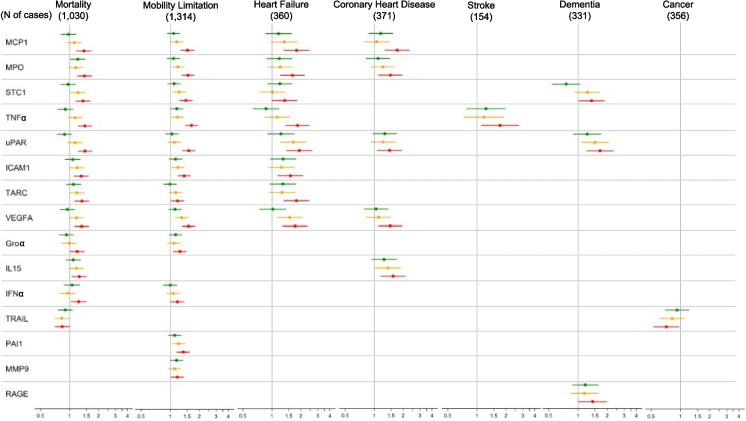
Forest plot of relative hazards for associations between senescence biomarkers and clinical outcomes, adjusted for age, sex, and race using the lowest quartile as the reference group. Only markers with statistically significant associations on the highest quartile are displayed

In summary, we noted that the highest levels of eighteen biomarkers were associated with significantly greater HRs for four or more of the six major health outcomes, besides cancer, during the follow-up period (Fig. 1). The highest quartiles of Eotaxin, IL8, OPN, PARC, Fas, MCP1, MPO, STC1, TNFα, and VEGFA were each associated with significantly increased risk for four health outcomes; the highest quartiles of TNFR1, Activin A, and µPAR were each associated with significantly increased risk for five health outcomes, and the highest quartile of GDF15, IL6, MMP1, MMP7, and TNFR2 were associated with significantly increased risk for all six major health outcomes, besides cancer.

### Senescence biomarkers as predictors of major health outcomes in older adults

To further examine the added value of senescence biomarkers as predictors of mortality, functional decline, and disease in older adults, we calculated concordance statistics (*C*-statistics). This approach enabled the comparison of the predictive performance of traditional demographic and clinical variables (i.e*.*, age, sex, and race) with and without the top biomarkers selected for each outcome through LASSO regression.

**Fig. 2:**
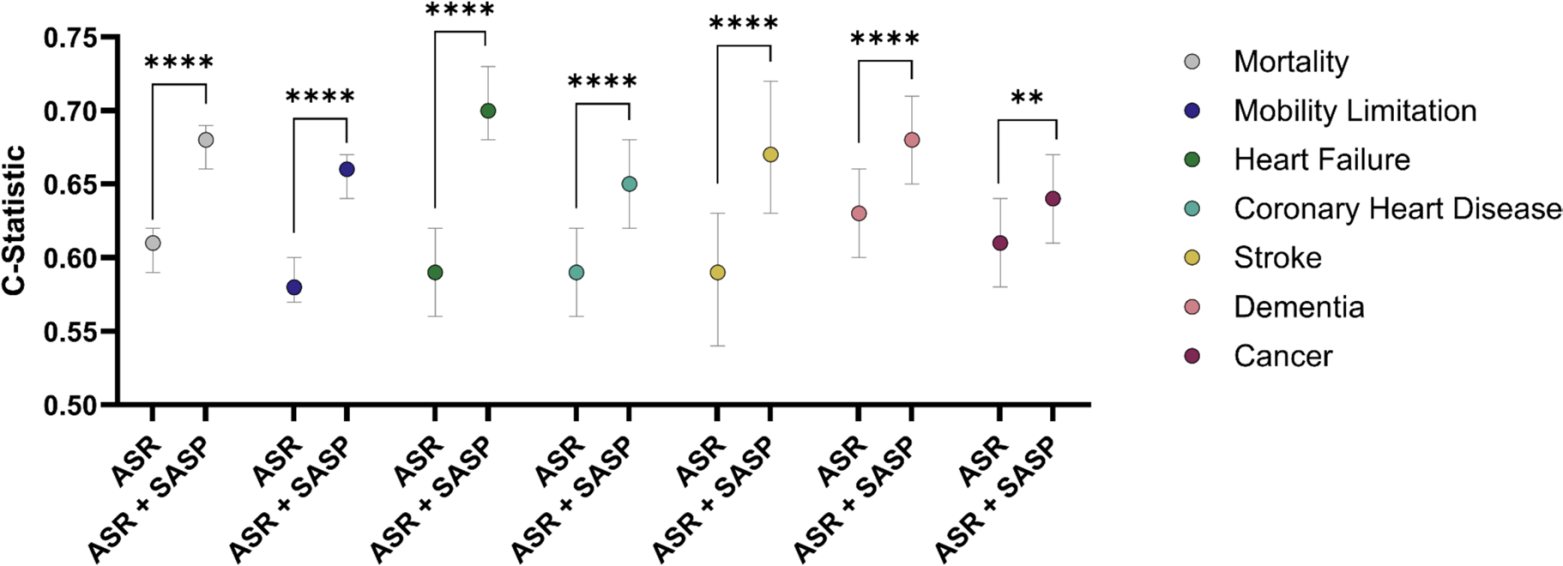
C-statistics comparing prediction of outcomes by age, sex, and race (ASR), SASP factors alone, and ASR plus SASP factors. SASP factors, selected by lasso regression, for each comparison are described in Supplemental Table S9–S15

The addition of senescence biomarkers increased the *C*-statistics from demographic and clinical variables alone for every outcome (Fig. 2 and Table 2). Addition of the top senescent biomarkers for the condition substantially and significantly increased the *C*-statistics for mortality, mobility limitation, heart failure, CHD, stroke, and dementia. The addition of the biomarkers made a statistically significant but small difference for cancer. Of note, for mortality, the *C*-statistics of the demographic and clinical variables alone was 0.61 and addition of the top 7 predictive biomarkers for mortality increased the *C*-statistics to 0.68 (Table 2). For mobility limitation, the addition of 5 senescence biomarkers increased the *C*-statistic from 0.58 to 0.66. Addition of GDF15, PARC, and MMP7 increased the *C*-statistic for heart failure from 0.59 to 0.70. For the occurrence of CHD, the *C*-statistics for the demographic and clinical variables was 0.59 that increased to 0.65 by the addition of only GDF15 and IL6. Similarly, for stroke the addition of only Eotaxin and IL6 increased the C-statistic from 0.59 to 0.67. Addition of GDF15 and MMP1 modestly increased the *C*-statistic for dementia from 0.63 to 0.68. The *C*-statistic for cancer increased little, from 0.61 to 0.64 by the addition of 4 biomarkers, MMP1, Activin A, TRAIL, and uPAR (Table 2). Of note, GDF15 was one of the top biomarkers for all conditions except stroke and cancer.

## Discussion

This prospective study of senescence biomarkers and major clinical health outcomes demonstrates that several proteins identified as common components of the SASP are associated with all-cause mortality, mobility limitation, HF, CHD, stroke, and dementia. The association of senescence biomarkers with a broad range of distinct age-related health outcomes fits the geroscience paradigm that fundamental mechanisms of aging are the root cause of most diseases.

The results support that the accumulation of senescent cells is a common denominator in the increased risk of human conditions that involve skeletal and cardiac muscle, vascular tissue, and the brain. All of the age-, race-, and gender-adjusted associations between senescence biomarkers and the clinical outcomes showed that increased serum protein concentrations were associated with increased risk. These data suggest that the measured factors, derived from various models of cell senescence, may reflect the burden of senescent cells in tissues. In multivariate models, there were only two outcomes for which lower levels of senescence biomarkers were associated with higher risk; lower serum concentrations of TRAIL were associated with greater risk of mortality, and lower levels of TRAIL and uPAR were associated with greater risk for cancer. These results may represent a chance finding arising from testing multiple associations, or be biologically plausible, e.g., senescence is a conserved anti-cancer mechanism.

The prospective nature of this study with a mean of 11**.**5 years of follow-up, and exclusion of participants with the health outcomes of interest as baseline, suggests that cellular senescence and the SASP are not merely a consequence of age-related diseases and functional decline but plausible mediators in humans. While additional research is needed to test this premise, the data from the current study complement mechanistic studies of senolytic and senomorphic interventions in a wide range of diseases modeled in mice that demonstrate therapeutic benefit of clearance of senescent cells and suppression of the SASP [4–10].

The significant associations between SASP factors and mortality include GDF15, VEGFA, PARC, and MMP2, that were also found to be associated with the risk of death in a cohort of patients from Mayo Clinic [16]. The proteins that were significantly associated with self-reported mobility disability in this study also overlap with several of those found to be associated with objective physical performance tests and endpoints in the LIFE study, which is consistent with our observation of strong associations between SASP factors and the risk of mobility limitation [15].

The association of senescence biomarkers with HF is supported by findings of increased cell senescence in cardiac muscle in older patients with this condition [4]. Similarly, the association between SASP factors and incident CHD is consistent with evidence that increasing senescence of arterial endothelial cells might play a role in its pathogenesis [4]. We found more limited associations between senescence biomarkers and incident stroke. This may reflect the smaller number of stroke cases in the analysis. Ischemic stroke may induce secretion of SASP [26], but evidence for a causal role of cell senescence in stroke is limited. The association with dementia is consistent with studies that have found senescent astrocytes, microglia, endothelial cells, and neurons in the brains of older people with Alzheimer’s disease (AD) and in animal models of ADL [27]. In mouse models, removing senescent cells reduces the amounts of β-amyloid peptide and tau-protein [27].

Elevated levels of senescence biomarkers were generally not associated with the risk of non-skin cancer. This is consistent with the general concept that senescence may protect against the development and progression of cancer [28]. However, as this outcome in HABC included diverse types of cancer, the study does not address potential associations with specific types of cancer.

The study has important strengths and limitations. Based on the large Health ABC cohort study, it has the statistical power to find associations with important aging-related clinical outcomes including mortality. It is a prospective study of a biracial cohort with similar numbers of women and men with a sufficient number of events and participants to find significant associations with important clinical endpoints. It is noted that the senescence cells are not the only source of the proteins measured in this study. The candidate senescence biomarkers were identified by exploiting the literature (Coppa and SenMayo) and using several experimental approaches, including studies of the secretome of several human cell types induced to senesce in vitro and the plasma of mice with high senescent cell burden (Refs. from “Methods”). Additional protein components and other types of molecules (e.g., metabolites and miRNA) produced by senescent cells in various tissues are likely to be discovered by the extensive SenNet project (https://sennetconsortium.org/). Confirmation that circulating concentrations of the proteins measured in this study, or factors that emerge in future studies, reflects the abundance of senescent cells in humans will require clinical trials of treatments with specific senolytic activity.

Overall, these results confirm biomarkers of cellular senescence are associated with mortality and the incidence of mobility disability in a diverse cohort of older adults and further suggest that are associated with several important aging-related clinical outcomes in humans. Emerging senolytic and senomorphic therapies may provide an innovative approach to mitigate the risk of these important conditions. Moreover, senescence biomarkers may offer utility in the identification of older adults most likely to benefit from such treatment and be useful in monitoring the efficacy of treatment in clinical trials.

## Supplementary Information

Below is the link to the electronic supplementary material.

Supplementary file1 (DOCX 687 KB)

## Data Availability

Data used for the study from Health ABC are available to investigators. Requests for access to the Health ABC data may be made to https://healthabc.nia.nih.gov/. Data used in the analyses are available by request from the first author.
