# Correction to: Biomarkers of cellular senescence and major health outcomes in older adults

**DOI:** 10.1007/s11357-025-01619-4

**Published:** 2025-03-26

**Authors:** Steven R. Cummings, Li‑Yung Lui, Zaira Aversa, Theresa Mau, Roger A. Fielding, Elizabeth J. Atkinson, Sheena Patel, Nathan LeBrasseur

**Affiliations:** 1 San Francisco Coordinating Center, California Pacific Medical Center Research Institute, San Francisco, CA USA; 2 Department of Epidemiology and Biostatistics, University of California, San Francisco, CA USA; 3 Robert and Arlene Kogod Center on Aging, Paul F. Glenn Center for the Biology of Aging Research, and Department of Physical Medicine and Rehabilitation, Mayo Clinic, Rochester, MN USA; 4 Metabolism and Basic Biology of Aging Directive, Jean Mayer USDA Human Nutrition Research Center on Aging, Tufts University, Medford, MA USA; 5 Department of Quantitative Health Sciences, Mayo Clinic, Rochester, MN USA

**Correction to: GeroScience**

10.1007/s11357-024-01474-9

In this article, the author’s name Zaira Aversa was incorrectly written as Aversa Zaira.

The author group and affiliation section has been updated.

