# Supplementary material for: Biomarkers of cellular senescence and major health outcomes in older adults
Source: GeroScience. 2024 Dec 18;47(3):3407–15. doi: 10.1007/s11357-024-01474-9 (PMC12181601; doi:10.1007/s11357-024-01474-9)

Supplementary information

Supplement Table S1

| **Alternative Name** | **Full Name** |
| --- | --- |
| Activin A | Activin A |
| ADAMTS13 | A disintegrin and metalloproteinase with thrombospondin motifs 13 |
| Eotaxin | Eotaxin |
| Fas | Tumor necrosis factor receptor superfamily member 6 |
| GDF15 | Growth/differentiation factor 15 |
| Groα | Growth-regulated alpha protein |
| ICAM1 | Intercellular adhesion molecule 1 |
| IFNα | Interferon alpha |
| IL15 | Interleukin 15 |
| IL6 | Interleukin 6 |
| IL7 | Interleukin 7 |
| IL8 | Interleukin 8 |
| MCP1 | Monocyte chemotactic protein 1 |
| MDC | Macrophage-derived chemokine |
| MMP1 | Matrix metalloproteinase 1 |
| MMP2 | Matrix metalloproteinase 2 |
| MMP7 | Matrix metalloproteinase 7 |
| MMP9 | Matrix metalloproteinase 9 |
| MPO | Myeloperoxidase |
| OPN | Osteopontin |
| PAI1 | Plasminogen activator inhibitor 1 |
| PARC | Pulmonary and activation-regulated chemokine |
| PDGF-AA | Platelet-derived growth factor alpha polypeptide a |
| PDGF-AB | Platelet-derived growth factor alpha polypeptide b |
| RAGE | Advanced glycosylation end product-specific receptor |
| RANTES | Regulated on Activation, Normal T Cell Expressed and Secreted |
| SOST | Sclerostin |
| STC1 | Stanniocalcin 1 |
| TARC | Thymus and activation-regulated chemokine |
| TNFα | Tumor necrosis factor alpha |
| TNFR1 | Tumor necrosis factor receptor 1 |
| TNFR2 | Tumor necrosis factor receptor 2 |
| TRAIL | Tumor necrosis factor-related apoptosis-inducing ligand |
| uPAR | Urokinase-type plasminogen activator receptor |
| VEGFA | Vascular endothelial growth factor A |

Supplement Table S2. Summary of associations between serum concentrations of senescence biomarkers and clinical outcome mortality (1030 cases) (adjusted for age, sex, and race). Associations expressed as hazard ratio (HR) and 95% confidence interval (CI) with Q1 as the reference group.

| Biomarkers |  |  | |  |  |
| --- | --- | --- | --- | --- | --- |
|  | Q1 | | Q2 | Q3 | Q4 |
| GDF15 | ref | 1.2 (1.0, 1.5) | | 1.6 (1.3, 1.9) | 2.6 (2.1, 3.1) |
| TNFR1 | ref | 1.0 (0.8, 1.2) | | 1.2 (1.0, 1.5) | 1.7 (1.4, 2.0) |
| IL6 | ref | 1.2 (1.0, 1.4) | | 1.4 (1.2, 1.7) | 1.7 (1.5, 2.1) |
| MMP1 | ref | 1.1 (0.9, 1.3) | | 1.3 (1.1, 1.6) | 1.6 (1.4, 2.0) |
| MMP7 | ref | 1.2 (1.0, 1.4) | | 1.2 (1.0, 1.4) | 1.6 (1.3, 1.9) |
| Activin A | ref | 1.2 (1.0, 1.4) | | 1.2 (1.0, 1.4) | 1.6 (1.3, 1.9) |
| TNFR2 | ref | 1.1 (0.9, 1.3) | | 1.2 (1.0, 1.5) | 1.6 (1.3, 1.9) |
| Eotaxin | ref | 1.0 (0.9, 1.3) | | 1.2 (1.0, 1.4) | 1.5 (1.3, 1.8) |
| IL8 | ref | 1.2 (1.0, 1.5) | | 1.3 (1.1, 1.5) | 1.5 (1.2, 1.7) |
| MDC | ref | 1.1 (0.9, 1.3) | | 1.1 (1.0, 1.4) | 1.5 (1.2, 1.7) |
| MMP2 | ref | 1.1 (0.9, 1.3) | | 1.3 (1.0, 1.5) | 1.5 (1.3, 1.8) |
| OPN | ref | 1.1 (0.9, 1.3) | | 1.2 (1.0, 1.5) | 1.5 (1.3, 1.8) |
| PARC | ref | 1.1 (0.9, 1.3) | | 1.2 (1.0, 1.5) | 1.5 (1.2, 1.8) |
| Fas | ref | 0.9 (0.7, 1.1) | | 1.0 (0.8, 1.2) | 1.4 (1.1, 1.6) |
| MCP1 | ref | 1.0 (0.8, 1.2) | | 1.1 (0.9, 1.3) | 1.4 (1.2, 1.7) |
| MPO | ref | 1.2 (1.0, 1.5) | | 1.2 (1.0, 1.4) | 1.4 (1.2, 1.7) |
| STC1 | ref | 1.0 (0.8, 1.2) | | 1.2 (1.0, 1.5) | 1.4 (1.2, 1.6) |
| TNFα | ref | 0.9 (0.8, 1.1) | | 1.1 (1.0, 1.4) | 1.4 (1.2, 1.7) |
| µPAR | ref | 0.9 (0.7, 1.1) | | 1.1 (1.0, 1.4) | 1.4 (1.2, 1.7) |
| ICAM1 | ref | 1.1 (0.9, 1.3) | | 1.2 (1.0, 1.4) | 1.3 (1.1, 1.6) |
| TARC | ref | 1.1 (0.9, 1.3) | | 1.2 (1.0, 1.4) | 1.3 (1.1, 1.6) |
| VEGFA | ref | 0.9 (0.8, 1.1) | | 1.2 (1.0, 1.4) | 1.3 (1.1, 1.6) |
| Groα | ref | 0.9 (0.8, 1.1) | | 1.0 (0.8, 1.2) | 1.2 (1.0, 1.4) |
| IL15 | ref | 1.1 (0.9, 1.3) | | 1.2 (1.0, 1.4) | 1.3 (1.1, 1.5) |
| IFNα | ref | 1.1 (0.9, 1.3) | | 1.0 (0.8, 1.2) | 1.2 (1.0, 1.5) |
| TRAIL | ref | 0.9 (0.8, 1.1) | | 0.8 (0.7, 1.0) | 0.8 (0.7, 1.0) |
| PAI1 | ref | 1.0 (0.9, 1.2) | | 1.0 (0.8, 1.2) | 1.1 (0.9, 1.3) |
| MMP9 | ref | 1.1 (0.9, 1.3) | | 1.1 (0.9, 1.3) | 1.1 (0.9, 1.3) |
| RAGE | ref | 1.0 (0.9, 1.2) | | 0.9 (0.8, 1.1) | 1.1 (0.9, 1.3) |
|  |  |  | |  |  |
| ADAMTS13 | ref | 0.9 (0.8, 1.1) | | 0.9 (0.8, 1.1) | 0.9 (0.8, 1.1) |
| IL7 | ref | 1.0 (0.8, 1.2) | | 1.1 (0.9, 1.3) | 1.1 (0.9, 1.3) |
| PDGF-AA | ref | 0.9 (0.8, 1.1) | | 1.1 (0.9, 1.3) | 1.0 (0.9, 1.2) |
| PDGF-AB | ref | 1.0 (0.8, 1.1) | | 1.0 (0.8, 1.1) | 1.1 (0.9, 1.3) |
| RANTES | ref | 1.0 (0.8, 1.1) | | 1.0 (0.9, 1.2) | 1.1 (0.9, 1.3) |
| SOST | ref | 1.1 (0.9, 1.3) | | 1.0 (0.8, 1.2) | 0.9 (0.7, 1.1) |

Supplement Table S3. Summary of associations between serum concentrations of senescence biomarkers and clinical outcome mobility limitation (1314 cases) adjusted for age, sex, and race. Associations expressed as hazard ratio (HR) and 95% confidence interval (CI) with Q1 as the reference group.

| Biomarkers |  |  | |  |  |
| --- | --- | --- | --- | --- | --- |
|  | Q1 | | Q2 | Q3 | Q4 |
| GDF15 | ref | 1.1 (0.9, 1.3) | | 1.4 (1.2, 1.7) | 2.1 (1.8, 2.5) |
| TNFR1 | ref | 1.2 (1.0, 1.3) | | 1.4 (1.2, 1.6) | 2.1 (1.8, 2.5) |
| IL6 | ref | 1.1 (1.0, 1.3) | | 1.6 (1.4, 1.9) | 1.6 (1.4, 1.9) |
| MMP1 | ref | 1.0 (0.9, 1.2) | | 1.2 (1.1, 1.4) | 1.4 (1.2, 1.7) |
| MMP7 | ref | 1.2 (1.0, 1.4) | | 1.1 (0.9, 1.3) | 1.5 (1.3, 1.8) |
| Activin A | ref | 1.0 (0.9, 1.2) | | 1.1 (1.0, 1.3) | 1.5 (1.3, 1.8) |
| TNFR2 | ref | 1.2 (1.0, 1.4) | | 1.3 (1.1, 1.5) | 1.7 (1.5, 2.0) |
| Eotaxin | ref | 1.1 (0.9, 1.3) | | 1.1 (0.9, 1.3) | 1.4 (1.2, 1.7) |
| IL8 | ref | 1.3 (1.1, 1.5) | | 1.3 (1.1, 1.6) | 1.3 (1.1, 1.6) |
| MDC | ref | 0.9 (0.8, 1.1) | | 1.0 (0.9, 1.2) | 1.3 (1.1, 1.5) |
| MMP2 | ref | 1.0 (0.9, 1.2) | | 1.2 (1.0, 1.4) | 1.2 (1.1, 1.5) |
| OPN | ref | 1.0 (0.9, 1.2) | | 1.0 (0.9, 1.2) | 1.4 (1.2, 1.7) |
| PARC | ref | 1.1 (1.0, 1.3) | | 1.4 (1.2, 1.6) | 1.6 (1.4, 1.9) |
| Fas | ref | 1.0 (0.9, 1.2) | | 1.1 (0.9, 1.2) | 1.4 (1.2, 1.6) |
| MCP1 | ref | 1.1 (0.9, 1.3) | | 1.2 (1.0, 1.4) | 1.5 (1.3, 1.8) |
| MPO | ref | 1.1 (0.9, 1.3) | | 1.2 (1.0, 1.4) | 1.5 (1.3, 1.8) |
| STC1 | ref | 1.1 (0.9, 1.3) | | 1.2 (1.1, 1.4) | 1.5 (1.2, 1.7) |
| TNFα | ref | 1.2 (1.0, 1.4) | | 1.2 (1.0, 1.4) | 1.7 (1.4, 1.9) |
| µPAR | ref | 1.0 (0.9, 1.2) | | 1.1 (0.9, 1.3) | 1.6 (1.3, 1.8) |
| ICAM1 | ref | 1.1 (1.0, 1.3) | | 1.2 (1.0, 1.4) | 1.4 (1.2, 1.6) |
| TARC | ref | 1.0 (0.9, 1.2) | | 1.1 (1.0, 1.3) | 1.2 (1.0, 1.4) |
| VEGFA | ref | 1.1 (1.0, 1.3) | | 1.3 (1.1, 1.5) | 1.5 (1.3, 1.8) |
| Groα | ref | 1.1 (1.0, 1.3) | | 1.1 (0.9, 1.3) | 1.3 (1.1, 1.5) |
| IL15 | ref | 1.0 (0.8, 1.1) | | 1.1 (0.9, 1.2) | 1.1 (1.0, 1.3) |
| IFNα | ref | 1.0 (0.8, 1.2) | | 1.1 (0.9, 1.3) | 1.2 (1.0, 1.4) |
| TRAIL | ref | 0.9 (0.8, 1.1) | | 0.9 (0.8, 1.0) | 0.9 (0.8, 1.1) |
| PAI1 | ref | 1.1 (1.0, 1.3) | | 1.2 (1.0, 1.4) | 1.4 (1.2, 1.6) |
| MMP9 | ref | 1.2 (1.0, 1.4) | | 1.1 (0.9, 1.3) | 1.2 (1.0, 1.4) |
| RAGE | ref | 1.0 (0.9, 1.2) | | 1.0 (0.9, 1.2) | 1.1 (0.9, 1.3) |
|  |  |  | |  |  |
| ADAMTS13 | ref | 1.0 (0.8, 1.2) | | 1.0 (0.9, 1.2) | 0.9 (0.8, 1.0) |
| IL7 | ref | 1.1 (0.9, 1.3) | | 1.0 (0.8, 1.1) | 1.1 (0.9, 1.2) |
| PDGF-AA | ref | 1.1 (0.9, 1.2) | | 1.0 (0.9, 1.2) | 1.1 (1.0, 1.3) |
| PDGF-AB | ref | 1.1 (0.9, 1.3) | | 1.1 (0.9, 1.3) | 1.1 (0.9, 1.3) |
| RANTES | ref | 1.1 (0.9, 1.3) | | 1.0 (0.8, 1.2) | 1.2 (1.0, 1.3) |
| SOST | ref | 1.1 (0.9, 1.2) | | 1.0 (0.8, 1.2) | 1.1 (0.9, 1.3) |

Supplement Table S4. Summary of associations between serum concentrations of senescence biomarkers and clinical outcome heart failure (360 cases) adjusted for age, sex, and race. Associations expressed as hazard ratio (HR) and 95% confidence interval (CI) with Q1 as the reference group.

| Biomarkers |  |  | |  |  |
| --- | --- | --- | --- | --- | --- |
|  | Q1 | | Q2 | Q3 | Q4 |
| GDF15 | ref | 1.3 (0.9, 1.8) | | 1.3 (1.0, 1.8) | 2.4 (1.8, 3.3) |
| TNFR1 | ref | 1.0 (0.7, 1.4) | | 1.3 (1.0, 1.8) | 2.1 (1.6, 2.9) |
| IL6 | ref | 1.3 (0.9, 1.8) | | 2.0 (1.5, 2.7) | 2.0 (1.4, 2.7) |
| MMP1 | ref | 1.4 (1.0, 1.9) | | 1.3 (1.0, 1.8) | 1.7 (1.3, 2.4) |
| MMP7 | ref | 1.4 (1.0, 1.9) | | 1.4 (1.0, 1.9) | 2.1 (1.5, 2.8) |
| Activin A | ref | 1.3 (0.9, 1.7) | | 1.1 (0.8, 1.6) | 1.8 (1.4, 2.5) |
| TNFR2 | ref | 0.9 (0.7, 1.3) | | 1.0 (0.7, 1.4) | 1.6 (1.2, 2.2) |
| Eotaxin | ref | 1.0 (0.7, 1.4) | | 1.1 (0.8, 1.5) | 1.4 (1.0, 1.8) |
| IL8 | ref | 1.3 (1.0, 1.8) | | 1.4 (1.0, 1.8) | 1.5 (1.1, 2.0) |
| MDC | ref | 0.7 (0.5, 0.9) | | 0.9 (0.7, 1.3) | 1.4 (1.0, 1.8) |
| MMP2 | ref | 1.1 (0.8, 1.4) | | 1.2 (0.9, 1.6) | 1.3 (1.0, 1.8) |
| OPN | ref | 1.0 (0.8, 1.4) | | 1.1 (0.8, 1.4) | 1.2 (0.9, 1.7) |
| PARC | ref | 1.5 (1.1, 2.1) | | 1.8 (1.3, 2.5) | 2.2 (1.6, 3.0) |
| Fas | ref | 1.0 (0.8, 1.4) | | 1.0 (0.7, 1.4) | 1.6 (1.2, 2.2) |
| MCP1 | ref | 1.2 (0.9, 1.6) | | 1.3 (1.0, 1.8) | 1.8 (1.3, 2.5) |
| MPO | ref | 1.2 (0.9, 1.6) | | 1.2 (0.9, 1.7) | 1.6 (1.2, 2.2) |
| STC1 | ref | 1.2 (0.9, 1.6) | | 1.0 (0.7, 1.4) | 1.4 (1.0, 1.8) |
| TNFα | ref | 0.9 (0.6, 1.2) | | 1.1 (0.8, 1.5) | 1.8 (1.4, 2.5) |
| µPAR | ref | 1.2 (0.9, 1.7) | | 1.7 (1.2, 2.3) | 1.9 (1.4, 2.6) |
| ICAM1 | ref | 1.3 (1.0, 1.8) | | 1.3 (0.9, 1.7) | 1.6 (1.1, 2.1) |
| TARC | ref | 1.3 (1.0, 1.8) | | 1.3 (0.9, 1.8) | 1.8 (1.3, 2.4) |
| VEGFA | ref | 1.0 (0.7, 1.4) | | 1.5 (1.1, 2.1) | 1.7 (1.3, 2.3) |
| Groα | ref | 1.1 (0.8, 1.5) | | 1.3 (1.0, 1.8) | 1.3 (0.9, 1.7) |
| IL15 | ref | 1.1 (0.8, 1.5) | | 1.4 (1.0, 1.8) | 1.3 (0.9, 1.7) |
| IFNα | ref | 1.2 (0.9, 1.7) | | 0.9 (0.7, 1.3) | 1.3 (0.9, 1.7) |
| TRAIL | ref | 0.8 (0.6, 1.1) | | 0.8 (0.6, 1.1) | 0.8 (0.6, 1.1) |
| PAI1 | ref | 1.0 (0.7, 1.3) | | 1.2 (0.9, 1.6) | 1.3 (0.9, 1.7) |
| MMP9 | ref | 1.1 (0.8, 1.5) | | 1.0 (0.7, 1.4) | 1.2 (0.9, 1.6) |
| RAGE | ref | 1.0 (0.7, 1.3) | | 1.1 (0.8, 1.5) | 1.1 (0.8, 1.5) |
|  |  |  | |  |  |
| ADAMTS13 | ref | 0.8 (0.6, 1.1) | | 0.9 (0.7, 1.2) | 0.8 (0.6, 1.1) |
| IL7 | ref | 0.9 (0.7, 1.2) | | 1.0 (0.8, 1.3) | 1.1 (0.8, 1.4) |
| PDGF-AA | ref | 1.0 (0.8, 1.4) | | 1.1 (0.8, 1.4) | 1.1 (0.8, 1.5) |
| PDGF-AB | ref | 1.3 (1.0, 1.8) | | 1.1 (0.8, 1.5) | 1.3 (0.9, 1.7) |
| RANTES | ref | 0.9 (0.7, 1.2) | | 0.9 (0.7, 1.2) | 1.1 (0.8, 1.4) |
| SOST | ref | 0.9 (0.7, 1.3) | | 1.0 (0.8, 1.4) | 1.0 (0.7, 1.4) |

Supplement Table S5. Summary of associations between serum concentrations of senescence biomarkers and clinical outcome coronary heart disease (371 cases) adjusted for age, sex, and race. Associations expressed as hazard ratio (HR) and 95% confidence interval (CI) with Q1 as the reference group.

| Biomarkers |  |  | |  |  |
| --- | --- | --- | --- | --- | --- |
|  | Q1 | | Q2 | Q3 | Q4 |
| GDF15 | ref | 0.9 (0.6, 1.2) | | 1.3 (1.0, 1.8) | 1.7 (1.3, 2.3) |
| TNFR1 | ref | 0.7 (0.5, 1.0) | | 1.2 (0.9, 1.6) | 1.2 (0.9, 1.6) |
| IL6 | ref | 1.2 (0.9, 1.7) | | 1.3 (1.0, 1.8) | 1.7 (1.3, 2.3) |
| MMP1 | ref | 1.2 (0.9, 1.7) | | 1.0 (0.8, 1.4) | 1.4 (1.0, 1.8) |
| MMP7 | ref | 1.0 (0.8, 1.4) | | 1.1 (0.8, 1.5) | 1.6 (1.2, 2.1) |
| Activin A | ref | 1.1 (0.8, 1.4) | | 1.3 (1.0, 1.8) | 1.6 (1.2, 2.2) |
| TNFR2 | ref | 0.9 (0.6, 1.2) | | 1.0 (0.7, 1.3) | 1.4 (1.1, 1.9) |
| Eotaxin | ref | 0.9 (0.7, 1.2) | | 0.9 (0.6, 1.2) | 1.1 (0.8, 1.5) |
| IL8 | ref | 1.5 (1.1, 2.0) | | 1.5 (1.1, 2.0) | 1.5 (1.1, 2.1) |
| MDC | ref | 0.8 (0.6, 1.1) | | 1.1 (0.8, 1.4) | 1.1 (0.8, 1.5) |
| MMP2 | ref | 0.7 (0.5, 0.9) | | 0.8 (0.6, 1.1) | 1.1 (0.8, 1.4) |
| OPN | ref | 1.0 (0.7, 1.3) | | 1.0 (0.7, 1.3) | 1.4 (1.1, 1.9) |
| PARC | ref | 1.3 (1.0, 1.8) | | 1.4 (1.0, 1.8) | 1.6 (1.2, 2.1) |
| Fas | ref | 1.0 (0.7, 1.3) | | 0.8 (0.6, 1.1) | 1.2 (0.9, 1.6) |
| MCP1 | ref | 1.2 (0.9, 1.6) | | 1.1 (0.8, 1.4) | 1.7 (1.3, 2.3) |
| MPO | ref | 1.1 (0.8, 1.5) | | 1.2 (0.9, 1.7) | 1.5 (1.1, 2.0) |
| STC1 | ref | 1.1 (0.8, 1.5) | | 1.0 (0.7, 1.3) | 1.2 (0.9, 1.5) |
| TNFα | ref | 0.9 (0.7, 1.2) | | 0.9 (0.7, 1.2) | 1.2 (0.9, 1.6) |
| µPAR | ref | 1.3 (1.0, 1.7) | | 1.2 (0.9, 1.7) | 1.4 (1.1, 2.0) |
| ICAM1 | ref | 1.1 (0.8, 1.5) | | 1.3 (1.0, 1.8) | 1.3 (1.0, 1.8) |
| TARC | ref | 0.8 (0.6, 1.1) | | 0.8 (0.6, 1.0) | 1.1 (0.8, 1.4) |
| VEGFA | ref | 1.0 (0.8, 1.4) | | 1.1 (0.8, 1.5) | 1.5 (1.1, 2.0) |
| Groα | ref | 1.3 (1.0, 1.7) | | 1.3 (1.0, 1.8) | 1.1 (0.8, 1.5) |
| IL15 | ref | 1.3 (0.9, 1.7) | | 1.4 (1.0, 1.9) | 1.6 (1.2, 2.1) |
| IFNα | ref | 0.8 (0.6, 1.1) | | 1.0 (0.7, 1.3) | 1.2 (0.9, 1.6) |
| TRAIL | ref | 0.9 (0.7, 1.2) | | 0.7 (0.5, 1.0) | 0.8 (0.6, 1.1) |
| PAI1 | ref | 1.0 (0.8, 1.4) | | 1.0 (0.7, 1.3) | 1.1 (0.9, 1.5) |
| MMP9 | ref | 1.0 (0.8, 1.4) | | 0.9 (0.7, 1.2) | 1.1 (0.8, 1.4) |
| RAGE | ref | 1.1 (0.8, 1.5) | | 1.2 (0.9, 1.7) | 1.1 (0.8, 1.5) |
|  |  |  | |  |  |
| ADAMTS13 | ref | 1.1 (0.8, 1.5) | | 1.0 (0.7, 1.3) | 1.0 (0.7, 1.3) |
| IL7 | ref | 0.9 (0.7, 1.2) | | 0.9 (0.7, 1.3) | 1.0 (0.7, 1.3) |
| PDGF-AA | ref | 0.7 (0.5, 0.9) | | 0.8 (0.6, 1.1) | 0.8 (0.6, 1.1) |
| PDGF-AB | ref | 0.9 (0.6, 1.2) | | 0.8 (0.6, 1.1) | 1.0 (0.8, 1.4) |
| RANTES | ref | 0.8 (0.6, 1.0) | | 0.8 (0.6, 1.1) | 0.9 (0.7, 1.2) |
| SOST | ref | 0.9 (0.7, 1.2) | | 0.9 (0.7, 1.2) | 1.1 (0.8, 1.5) |

Supplement Table S6. Summary of associations between serum concentrations of senescence biomarkers and clinical outcome stroke (154 cases) adjusted for age, sex, and race. Associations expressed as hazard ratio (HR) and 95% confidence interval (CI) with Q1 as the reference group.

| Biomarkers |  |  | |  |  |
| --- | --- | --- | --- | --- | --- |
|  | Q1 | | Q2 | Q3 | Q4 |
| GDF15 | ref | 1.4 (0.8, 2.2) | | 1.7 (1.1, 2.8) | 2.2 (1.4, 3.6) |
| TNFR1 | ref | 1.7 (1.1, 2.8) | | 1.5 (0.9, 2.5) | 2.3 (1.4, 3.7) |
| IL6 | ref | 1.3 (0.8, 2.1) | | 1.7 (1.1, 2.7) | 1.8 (1.2, 2.9) |
| MMP1 | ref | 1.6 (1.0, 2.5) | | 1.5 (0.9, 2.5) | 2.0 (1.2, 3.2) |
| MMP7 | ref | 1.0 (0.6, 1.7) | | 1.1 (0.7, 1.8) | 1.7 (1.1, 2.6) |
| Activin A | ref | 1.2 (0.8, 2.0) | | 1.4 (0.9, 2.2) | 1.3 (0.8, 2.2) |
| TNFR2 | ref | 1.5 (0.9, 2.3) | | 1.1 (0.7, 1.8) | 1.6 (1.0, 2.6) |
| Eotaxin | ref | 1.6 (1.0, 2.6) | | 1.0 (0.6, 1.7) | 2.1 (1.3, 3.4) |
| IL8 | ref | 1.2 (0.7, 1.9) | | 1.3 (0.8, 2.0) | 1.5 (1.0, 2.4) |
| MDC | ref | 1.0 (0.6, 1.6) | | 0.9 (0.6, 1.5) | 1.3 (0.8, 2.0) |
| MMP2 | ref | 1.3 (0.8, 2.1) | | 1.6 (1.0, 2.5) | 1.8 (1.1, 2.9) |
| OPN | ref | 0.7 (0.4, 1.1) | | 0.9 (0.6, 1.4) | 1.0 (0.7, 1.6) |
| PARC | ref | 0.7 (0.5, 1.1) | | 0.8 (0.5, 1.2) | 0.8 (0.5, 1.3) |
| Fas | ref | 1.1 (0.7, 1.8) | | 1.2 (0.8, 2.0) | 1.3 (0.8, 2.2) |
| MCP1 | ref | 0.9 (0.5, 1.4) | | 1.1 (0.7, 1.7) | 1.1 (0.7, 1.8) |
| MPO | ref | 1.1 (0.7, 1.8) | | 1.2 (0.8, 2.0) | 1.3 (0.8, 2.1) |
| STC1 | ref | 1.0 (0.7, 1.6) | | 1.2 (0.8, 1.9) | 1.0 (0.6, 1.6) |
| TNFα | ref | 1.2 (0.8, 2.0) | | 1.2 (0.7, 1.9) | 1.7 (1.1, 2.7) |
| µPAR | ref | 1.0 (0.6, 1.5) | | 1.3 (0.8, 2.0) | 1.4 (0.9, 2.2) |
| ICAM1 | ref | 1.0 (0.7, 1.6) | | 1.0 (0.6, 1.6) | 1.2 (0.7, 1.8) |
| TARC | ref | 0.8 (0.5, 1.3) | | 1.2 (0.7, 1.8) | 1.0 (0.6, 1.6) |
| VEGFA | ref | 1.4 (0.9, 2.2) | | 1.2 (0.7, 1.9) | 1.2 (0.8, 2.0) |
| Groα | ref | 1.1 (0.7, 1.8) | | 1.6 (1.0, 2.5) | 1.1 (0.7, 1.8) |
| IL15 | ref | 0.7 (0.4, 1.1) | | 0.7 (0.4, 1.1) | 0.8 (0.5, 1.3) |
| IFNα | ref | 0.9 (0.5, 1.5) | | 1.3 (0.8, 2.1) | 1.1 (0.7, 1.8) |
| TRAIL | ref | 1.1 (0.7, 1.7) | | 0.6 (0.3, 1.0) | 1.1 (0.7, 1.7) |
| PAI1 | ref | 1.2 (0.8, 1.9) | | 1.0 (0.6, 1.6) | 1.4 (0.9, 2.2) |
| MMP9 | ref | 1.1 (0.7, 1.8) | | 1.5 (0.9, 2.3) | 1.5 (1.0, 2.4) |
| RAGE | ref | 0.9 (0.6, 1.4) | | 1.2 (0.8, 1.9) | 1.0 (0.6, 1.6) |
|  |  |  | |  |  |
| ADAMTS13 | ref | 1.3 (0.8, 2.1) | | 1.5 (1.0, 2.4) | 1.2 (0.8, 2.0) |
| IL7 | ref | 0.8 (0.5, 1.2) | | 1.0 (0.7, 1.5) | 0.8 (0.5, 1.2) |
| PDGF-AA | ref | 0.8 (0.5, 1.3) | | 1.1 (0.7, 1.8) | 1.2 (0.8, 1.8) |
| PDGF-AB | ref | 0.6 (0.4, 1.0) | | 1.0 (0.6, 1.5) | 0.9 (0.6, 1.4) |
| RANTES | ref | 0.9 (0.6, 1.4) | | 1.0 (0.7, 1.6) | 1.1 (0.7, 1.7) |
| SOST | ref | 0.8 (0.5, 1.3) | | 0.9 (0.5, 1.4) | 1.0 (0.6, 1.6) |

Supplement Table S7. Summary of associations between serum concentrations of senescence biomarkers and clinical outcome dementia (331 cases) adjusted for (age, sex, and race. Associations expressed as hazard ratio (HR) and 95% confidence interval (CI) with Q1 as the reference group.

| Biomarkers |  |  | |  |  |
| --- | --- | --- | --- | --- | --- |
|  | Q1 | | Q2 | Q3 | Q4 |
| GDF15 | ref | 0.9 (0.7, 1.3) | | 1.5 (1.1, 2.1) | 2.3 (1.7, 3.2) |
| TNFR1 | ref | 1.2 (0.9, 1.7) | | 1.2 (0.9, 1.7) | 1.9 (1.4, 2.6) |
| IL6 | ref | 0.9 (0.7, 1.2) | | 1.2 (0.9, 1.6) | 1.5 (1.1, 2.0) |
| MMP1 | ref | 1.5 (1.1, 2.0) | | 1.2 (0.8, 1.6) | 1.7 (1.2, 2.3) |
| MMP7 | ref | 1.1 (0.8, 1.5) | | 1.2 (0.9, 1.7) | 1.6 (1.2, 2.3) |
| Activin A | ref | 1.0 (0.8, 1.4) | | 1.1 (0.8, 1.5) | 1.5 (1.1, 2.0) |
| TNFR2 | ref | 0.9 (0.6, 1.2) | | 1.2 (0.9, 1.6) | 1.5 (1.1, 2.0) |
| Eotaxin | ref | 1.1 (0.8, 1.5) | | 1.1 (0.8, 1.6) | 1.4 (1.0, 1.8) |
| IL8 | ref | 1.1 (0.8, 1.5) | | 1.1 (0.8, 1.6) | 1.3 (0.9, 1.8) |
| MDC | ref | 0.8 (0.6, 1.1) | | 0.9 (0.7, 1.3) | 0.8 (0.6, 1.1) |
| MMP2 | ref | 0.9 (0.7, 1.3) | | 1.0 (0.7, 1.4) | 1.3 (1.0, 1.8) |
| OPN | ref | 1.2 (0.9, 1.7) | | 1.3 (1.0, 1.8) | 1.4 (1.0, 1.9) |
| PARC | ref | 1.0 (0.7, 1.3) | | 1.1 (0.8, 1.5) | 1.1 (0.8, 1.5) |
| Fas | ref | 1.0 (0.7, 1.4) | | 1.4 (1.0, 1.9) | 1.4 (1.0, 2.0) |
| MCP1 | ref | 0.8 (0.6, 1.1) | | 0.9 (0.7, 1.2) | 1.2 (0.9, 1.7) |
| MPO | ref | 1.1 (0.8, 1.4) | | 0.9 (0.6, 1.2) | 1.1 (0.8, 1.4) |
| STC1 | ref | 0.8 (0.5, 1.1) | | 1.3 (0.9, 1.7) | 1.4 (1.0, 1.9) |
| TNFα | ref | 0.8 (0.6, 1.1) | | 1.2 (0.9, 1.6) | 1.3 (1.0, 1.8) |
| µPAR | ref | 1.2 (0.9, 1.7) | | 1.5 (1.1, 2.1) | 1.7 (1.2, 2.3) |
| ICAM1 | ref | 0.8 (0.6, 1.1) | | 1.1 (0.8, 1.4) | 1.2 (0.9, 1.6) |
| TARC | ref | 1.2 (0.9, 1.6) | | 0.9 (0.6, 1.2) | 1.1 (0.8, 1.5) |
| VEGFA | ref | 1.2 (0.9, 1.6) | | 1.1 (0.8, 1.5) | 1.2 (0.9, 1.7) |
| Groα | ref | 1.0 (0.7, 1.4) | | 1.1 (0.8, 1.5) | 1.2 (0.9, 1.6) |
| IL15 | ref | 0.8 (0.6, 1.1) | | 1.0 (0.7, 1.4) | 1.1 (0.8, 1.5) |
| IFNα | ref | 1.4 (1.0, 1.9) | | 1.0 (0.7, 1.5) | 1.3 (0.9, 1.8) |
| TRAIL | ref | 1.1 (0.8, 1.5) | | 1.0 (0.7, 1.3) | 1.0 (0.7, 1.3) |
| PAI1 | ref | 1.1 (0.8, 1.5) | | 1.0 (0.7, 1.3) | 1.0 (0.7, 1.3) |
| MMP9 | ref | 0.9 (0.7, 1.3) | | 0.9 (0.7, 1.2) | 0.8 (0.6, 1.0) |
| RAGE | ref | 1.2 (0.9, 1.6) | | 1.2 (0.8, 1.6) | 1.4 (1.0, 2.0) |
|  |  |  | |  |  |
| ADAMTS13 | ref | 1.0 (0.7, 1.3) | | 1.2 (0.9, 1.6) | 1.1 (0.8, 1.5) |
| IL7 | ref | 1.0 (0.8, 1.4) | | 1.0 (0.8, 1.4) | 1.1 (0.8, 1.5) |
| PDGF-AA | ref | 1.0 (0.7, 1.4) | | 1.1 (0.8, 1.5) | 1.1 (0.8, 1.5) |
| PDGF-AB | ref | 1.0 (0.7, 1.3) | | 1.1 (0.8, 1.5) | 1.1 (0.8, 1.4) |
| RANTES | ref | 0.9 (0.7, 1.3) | | 1.1 (0.8, 1.5) | 1.1 (0.8, 1.5) |
| SOST | ref | 0.9 (0.7, 1.2) | | 0.7 (0.5, 1.0) | 1.0 (0.7, 1.3) |

Supplement Table S8. Summary of associations between serum concentrations of senescence biomarkers and clinical outcome incident cancer (356 cases) adjusted for age, sex, and race. Associations expressed as hazard ratio (HR) and 95% confidence interval (CI) with Q1 as the reference group.

| Biomarkers |  |  | |  |  |
| --- | --- | --- | --- | --- | --- |
|  | Q1 | | Q2 | Q3 | Q4 |
| GDF15 | ref | 1.1 (0.8, 1.5) | | 1.0 (0.8, 1.4) | 1.3 (0.9, 1.7) |
| TNFR1 | ref | 1.1 (0.9, 1.5) | | 1.2 (0.9, 1.7) | 1.4 (1.0, 1.8) |
| IL6 | ref | 1.0 (0.7, 1.3) | | 1.1 (0.8, 1.4) | 1.0 (0.8, 1.4) |
| MMP1 | ref | 1.5 (1.1, 2.1) | | 1.9 (1.4, 2.5) | 1.7 (1.2, 2.3) |
| MMP7 | ref | 1.1 (0.8, 1.5) | | 1.0 (0.8, 1.4) | 1.0 (0.7, 1.4) |
| Activin A | ref | 1.0 (0.8, 1.4) | | 1.3 (0.9, 1.7) | 1.4 (1.0, 1.9) |
| TNFR2 | ref | 0.9 (0.6, 1.2) | | 1.2 (0.9, 1.6) | 1.2 (0.9, 1.6) |
| Eotaxin | ref | 1.0 (0.7, 1.3) | | 0.9 (0.6, 1.2) | 1.1 (0.8, 1.4) |
| IL8 | ref | 0.9 (0.7, 1.2) | | 0.9 (0.7, 1.2) | 1.1 (0.8, 1.4) |
| MDC | ref | 1.2 (0.9, 1.6) | | 1.2 (0.9, 1.6) | 1.3 (0.9, 1.8) |
| MMP2 | ref | 1.0 (0.8, 1.4) | | 0.9 (0.7, 1.3) | 0.9 (0.6, 1.2) |
| OPN | ref | 1.3 (1.0, 1.7) | | 1.1 (0.8, 1.4) | 1.4 (1.0, 1.9) |
| PARC | ref | 1.0 (0.8, 1.4) | | 1.2 (0.9, 1.6) | 1.2 (0.9, 1.6) |
| Fas | ref | 0.9 (0.7, 1.2) | | 1.1 (0.8, 1.5) | 1.2 (0.9, 1.6) |
| MCP1 | ref | 1.1 (0.8, 1.5) | | 1.0 (0.7, 1.4) | 1.0 (0.7, 1.4) |
| MPO | ref | 0.9 (0.7, 1.2) | | 1.0 (0.7, 1.3) | 1.2 (0.9, 1.6) |
| STC1 | ref | 1.0 (0.8, 1.4) | | 0.9 (0.7, 1.3) | 1.1 (0.8, 1.5) |
| TNFα | ref | 1.0 (0.7, 1.3) | | 0.9 (0.6, 1.2) | 1.3 (0.9, 1.7) |
| µPAR | ref | 0.7 (0.5, 1.0) | | 0.9 (0.7, 1.2) | 0.8 (0.6, 1.1) |
| ICAM1 | ref | 0.8 (0.6, 1.1) | | 1.0 (0.7, 1.3) | 0.9 (0.7, 1.2) |
| TARC | ref | 1.0 (0.8, 1.4) | | 1.0 (0.8, 1.4) | 1.0 (0.7, 1.4) |
| VEGFA | ref | 1.3 (1.0, 1.8) | | 1.3 (0.9, 1.7) | 1.2 (0.9, 1.7) |
| Groα | ref | 1.1 (0.8, 1.4) | | 1.1 (0.8, 1.5) | 1.1 (0.8, 1.5) |
| IL15 | ref | 1.1 (0.8, 1.4) | | 1.0 (0.7, 1.4) | 1.2 (0.9, 1.6) |
| IFNα | ref | 1.2 (0.9, 1.6) | | 1.2 (0.9, 1.7) | 1.0 (0.7, 1.4) |
| TRAIL | ref | 0.9 (0.7, 1.2) | | 0.8 (0.6, 1.1) | 0.7 (0.5, 1.0) |
| PAI1 | ref | 1.1 (0.8, 1.5) | | 1.2 (0.9, 1.6) | 1.1 (0.8, 1.4) |
| MMP9 | ref | 1.3 (1.0, 1.8) | | 1.5 (1.1, 2.1) | 1.1 (0.8, 1.5) |
| RAGE | ref | 1.1 (0.8, 1.4) | | 0.8 (0.6, 1.1) | 0.8 (0.6, 1.1) |
|  |  |  | |  |  |
| ADAMTS13 | ref | 0.9 (0.6, 1.1) | | 0.8 (0.6, 1.1) | 0.9 (0.7, 1.2) |
| IL7 | ref | 1.0 (0.8, 1.4) | | 1.1 (0.8, 1.5) | 0.9 (0.7, 1.2) |
| PDGF-AA | ref | 1.1 (0.8, 1.5) | | 1.5 (1.1, 2.0) | 1.1 (0.8, 1.5) |
| PDGF-AB | ref | 1.1 (0.8, 1.5) | | 1.1 (0.8, 1.5) | 1.0 (0.7, 1.4) |
| RANTES | ref | 1.2 (0.9, 1.6) | | 1.3 (1.0, 1.7) | 0.9 (0.7, 1.2) |
| SOST | ref | 1.2 (0.9, 1.6) | | 1.3 (0.9, 1.8) | 1.3 (0.9, 1.7) |

Supplement S9. Hazard ratios (HR) from Cox regression for mortality for variables selected using Lasso

| Characteristic | Potential predictors include age, sex, and race | Potential predictors include all biomarkers* | Potential predictors include all biomarkers* with age, sex, and race forced in |
| --- | --- | --- | --- |
|  | **HR** | **HR** | **HR** |
| Age (5-year increase) | 1.57 | - | 1.37 |
| Sex (men vs. women) | 1.53 | - | 1.34 |
| Race (white vs. non-white) | 0.71 | - | 0.82 |
| Senescence biomarkers  (Q4 vs. Q1) |  |  |  |
| GDF15 | - | 2.14 | 1.96 |
| IL6 | - | 1.42 | 1.37 |
| MMP1 | - | 1.28 | 1.31 |
| MMP7 | - | 1.28 | 1.25 |
| Activin A | - | 1.22 | 1.23 |
| Eotaxin | - | 1.43 | 1.37 |
| MMP2 | - | 1.27 | 1.18 |
| OPN | - | 1.24 | 1.17 |
| TRAIL | - | 0.62 | 0.65 |
| PARC | - | 1.10 | 1.06 |
| MDC | - | 1.04 | 1.11 |
| IL8 | - | 1.18 | 1.13 |
| uPAR | - | 0.90 | 0.95 |
| TNFα | - | 0.95 | 0.98 |
| MCP1 | - | 1.01 | 0.94 |
| STC1 | - | 1.14 | 1.11 |
| TARC | - | 1.12 | 1.06 |
| ICAM1 | - | 1.11 | 1.12 |
| IL15 | - | 1.00 | 0.99 |
| Gro-alpha | - | 0.98 | 0.97 |
| C-statistic (95% CI) | 0.61 (0.59, 0.62) | 0.67 (0.65, 0.68) | 0.68 (0.66, 0.69) |

Supplement S10. Hazard ratios (HR) from Cox regression for mobility disability for variables selected using Lasso

| Characteristic | Potential predictors include age, sex, and race | Potential predictors include all biomarkers* | Potential predictors include all biomarkers* with age, sex, and race forced in |
| --- | --- | --- | --- |
|  | **HR** | **HR** | **HR** |
| Age (5-year increase) | 1.30 | - | 1.18 |
| Sex (men vs. women) | 0.88 | - | 0.74 |
| Race (white vs. non-white) | 0.69 | - | 0.80 |
| Senescence biomarkers  (Q4 vs. Q1) |  |  |  |
| TNFR1 | - | 1.31 | 1.40 |
| GDF15 | - | 1.37 | 1.39 |
| TNFα | - | 1.07 | 1.08 |
| IL6 | - | 1.25 | 1.23 |
| PARC | - | 1.26 | 1.20 |
| uPAR | - | 1.01 | 0.96 |
| MMP7 | - | 1.26 | 1.17 |
| MPO | - | 1.24 | 1.23 |
| MCP1 | - | 1.11 | 1.05 |
| STC1 | - | 1.12 | 1.13 |
| OPN | - | 1.18 | 1.20 |
| MMP1 | - | 1.16 | 1.18 |
| Eotaxin | - | 1.19 | 1.22 |
| IFN-alpha | - | 1.06 | 1.05 |
| ICAM1 | - | 1.01 | 1.13 |
| Fas | - | 0.78 | 0.85 |
| PAI1 | - | 0.98 | 1.01 |
| IL8 | - | 1.14 | 1.10 |
| MMP2 | - | 1.09 | 1.01 |
| C-statistic (95% CI) | 0.58 (0.57, 0.60) | 0.64 (0.62, 0.66) | 0.66 (0.64, 0.67) |

Supplement S11. Hazard ratios (HR) from Cox regression for heart failure for variables selected using Lasso

| Characteristic | Potential predictors include age, sex, and race | Potential predictors include all biomarkers* | Potential predictors include all biomarkers* with age, sex, and race forced in |
| --- | --- | --- | --- |
|  | **HR** | **HR** | **HR** |
| Age (5-year increase) | 1.61 | - | 1.43 |
| Sex (men vs. women) | 1.36 | - | 1.20 |
| Race (white vs. non-white) | 0.79 | - | 0.95 |
| Senescence biomarkers  (Q4 vs. Q1) |  |  |  |
| GDF15 | - | 1.61 | 1.47 |
| PARC | - | 1.65 | 1.65 |
| MMP7 | - | 1.62 | 1.61 |
| TNFR1 | - | 1.41 | 1.39 |
| IL6 | - | 1.38 | 1.35 |
| Activin A | - | 1.39 | 1.31 |
| TARC | - | 1.35 | 1.32 |
| TNFα | - | 1.31 | 1.33 |
| uPAR | - | 1.22 | 1.24 |
| MCP1 | - | 1.20 | 1.17 |
| ICAM1 | - | 1.21 | 1.20 |
| MPO | - | 1.15 | 1.14 |
| MMP1 | - | 1.14 | 1.11 |
| Eotaxin | - | 1.13 | 1.07 |
| VEGFA | - | 0.99 | 1.10 |
| TNFR2 | - | 0.75 | 0.74 |
| Fas | - | 0.84 | 0.78 |
| IL15 | - | 0.88 | 0.86 |
| MDC | - | 0.84 | 0.87 |
| STC1 | - | 0.97 | 0.98 |
| C-statistic (95% CI) | 0.59 (0.56, 0.62) | 0.70 (0.67, 0.72) | 0.70 (0.68, 0.73) |

Supplement S12. Hazard ratios (HR) from Cox regression for coronary heart disease for variables selected using Lasso

| Characteristic | Potential predictors include age, sex, and race | Potential predictors include all biomarkers* | Potential predictors include all biomarkers* with age, sex, and race forced in |
| --- | --- | --- | --- |
|  | **HR** | **HR** | **HR** |
| Age (5-year increase) | 1.18 | - | 1.08 |
| Sex (men vs. women) | 1.95 | - | 1.79 |
| Race (white vs. non-white) | 1.02 | - | 1.16 |
| Senescence biomarkers  (Q4 vs. Q1) |  |  |  |
| GDF15 | - | 1.68 | 1.44 |
| IL6 | - | 1.37 | 1.41 |
| MCP1 | - | 1.27 | 1.29 |
| Activin A | - | 1.42 | 1.35 |
| PARC | - | 1.35 | 1.34 |
| MPO | - | 1.20 | 1.22 |
| uPAR | - | 0.97 | 1.04 |
| C-statistic (95% CI) | 0.59 (0.56, 0.62) | 0.63 (0.60, 0.66) | 0.65 (0.62, 0.68) |

Supplement S13. Hazard ratios (HR) from Cox regression for stroke for variables selected using Lasso

| Characteristic | Potential predictors include age, sex, and race | Potential predictors include all biomarkers* | Potential predictors include all biomarkers* with age, sex, and race forced in |
| --- | --- | --- | --- |
|  | **HR** | **HR** | **HR** |
| Age (5-year increase) | 1.27 | - | 1.10 |
| Sex (men vs. women) | 1.13 | - | 0.99 |
| Race (white vs. non-white) | 0.54 | - | 0.61 |
| Senescence biomarkers  (Q4 vs. Q1) |  |  |  |
| IL6 | - | 1.90 | 1.68 |
| Eotaxin | - | 1.82 | 1.69 |
| GDF15 | - | 1.77 | 1.61 |
| MMP2 | - | 1.65 | 1.54 |
| MMP1 | - | 1.53 | 1.53 |
| TNFR1 | - | 1.14 | 1.40 |
| C-statistic (95% CI) | 0.59 (0.54, 0.63) | 0.66 (0.62, 0.71) | 0.67 (0.63, 0.72) |

Supplement S14. Hazard ratios (HR) from Cox regression for dementia for variables selected using Lasso

| Characteristic | Potential predictors include age, sex, and race | Potential predictors include all biomarkers* | Potential predictors include all biomarkers* with age, sex, and race forced in |
| --- | --- | --- | --- |
|  | **HR** | **HR** | **HR** |
| Age (5-year increase) | 2.04 | - | 1.87 |
| Sex (men vs. women) | 0.99 | - | 0.86 |
| Race (white vs. non-white) | 0.74 | - | 0.80 |
| Senescence biomarkers  (Q4 vs. Q1) |  |  |  |
| GDF15 | - | 2.03 | 1.86 |
| MMP1 | - | 1.51 | 1.53 |
| uPAR | - | 1.18 | 1.11 |
| MMP7 | - | 1.30 | 1.14 |
| IL6 | - | 1.25 | 1.24 |
| TNFR2 | - | 0.99 | 0.98 |
| OPN | - | 1.21 | 1.15 |
| RAGE | - | 1.05 | 1.22 |
| STC1 | - | 1.01 | 1.05 |
| C-statistic (95% CI) | 0.63 (0.60, 0.66) | 0.65 (0.62, 0.68) | 0.68 (0.65, 0.71) |

Supplement S15. Hazard ratios (HR) from Cox regression for cancer for variables selected using Lasso

| Characteristic | Potential predictors include age, sex, and race | Potential predictors include all biomarkers* | Potential predictors include all biomarkers* with age, sex, and race forced in |
| --- | --- | --- | --- |
|  | **HR** | **HR** | **HR** |
| Age (5-year increase) | 1.13 | - | 1.10 |
| Sex (men vs. women) | 2.01 | - | 1.91 |
| Race (white vs. non-white) | 0.72 | - | 0.72 |
| Senescence biomarkers  (Q4 vs. Q1) |  |  |  |
| MMP1 | - | 1.98 | 1.94 |
| Activin A | - | 1.57 | 1.45 |
| TRAIL |  | 0.64 | 0.63 |
| uPAR | - | 0.64 | 0.69 |
| C-statistic (95% CI) | 0.61 (0.58, 0.64) | 0.60 (0.57, 0.63) | 0.64 (0.61, 0.67) |

Supplemental Figure 1. Spearman correlations between serum concentrations of factors.


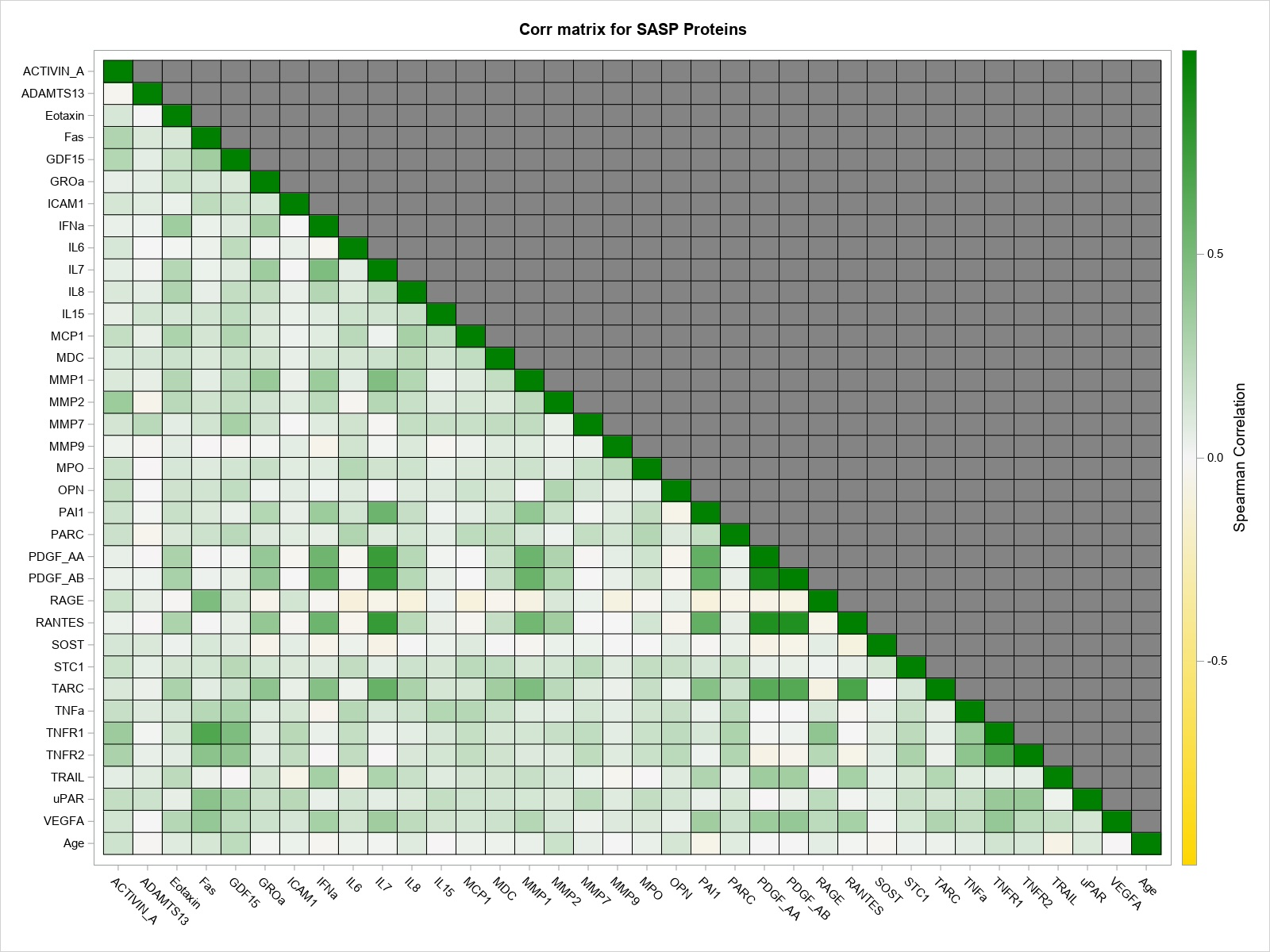

Supplement: Supplementary file 1 — Supplementary file1 (DOCX 687 KB) [file 11357_2024_1474_MOESM1_ESM.docx]
